# Selection of Candidate Genes Conferring Blast Resistance and Heat Tolerance in Rice through Integration of Meta-QTLs and RNA-Seq

**DOI:** 10.3390/genes13020224

**Published:** 2022-01-25

**Authors:** Tian Tian, Lijuan Chen, Yufang Ai, Huaqin He

**Affiliations:** College of Life Sciences, Fujian Agriculture and Forestry University, Fuzhou 350002, China; tiantian@m.fafu.edu.cn (T.T.); chenlj136@gmail.com (L.C.)

**Keywords:** rice (*Oryza sativa* L.), *Magnaporthe oryzae*, high temperature, QTL, meta-analysis, RNA-seq, candidate genes

## Abstract

Due to global warming, high temperature is a significant environmental stress for rice production. Rice (*Oryza sativa* L.), one of the most crucial cereal crops, is also seriously devastated by *Magnaporthe oryzae*. Therefore, it is essential to breed new rice cultivars with blast and heat tolerance. Although progress had been made in QTL mapping and RNA-seq analysis in rice in response to blast and heat stresses, there are few reports on simultaneously mining blast-resistant and heat-tolerant genes. In this study, we separately conducted meta-analysis of 839 blast-resistant and 308 heat-tolerant QTLs in rice. Consequently, 7054 genes were identified in 67 blast-resistant meta-QTLs with an average interval of 1.00 Mb. Likewise, 6425 genes were obtained in 40 heat-tolerant meta-QTLs with an average interval of 1.49 Mb. Additionally, using differentially expressed genes (DEGs) in the previous research and GO enrichment analysis, 55 DEGs were co-located on the common regions of 16 blast-resistant and 14 heat-tolerant meta-QTLs. Among, *OsChib3H-c*, *OsJAMyb*, *Pi-k*, *OsWAK1*, *OsMT2b*, *OsTPS3*, *OsHI-LOX*, *OsACLA-2* and *OsGS2* were the significant candidate genes to be further investigated. These results could provide the gene resources for rice breeding with excellent resistance to these 2 stresses, and help to understand how plants response to the combination stresses of blast fungus and high temperature.

## 1. Introduction

Rice is a significant staple food worldwide that provides more than 20% of the daily caloric needs for at least 50% of the global population [1]. To meet the demand of a growing global population, rice yields need a yearly increase of 0.6 to 0.9% [2]. However, rice frequently suffers from various biotic and abiotic stresses in nature [3,4]. As a consequence of global warming, the combination of pathogens and high temperatures (HT) frequently exists in the cultivation of cereal crops [5,6,7,8]. As the “cancer” of rice, *M. oryzae* is widely distributed, and terribly destructive under favorable conditions, which can cause severe yield losses in rice [9,10,11]. It is estimated that yearly, the rice blast fungus can destroy enough rice to feed 60 million people [12]. Likewise, HT also poses a serious threat to rice growth and development that harshly affects rice yields and quality [13,14]. Currently, introgression of the resistant and tolerant genes has been proven to improve resistance and tolerance in existing rice cultivars [15,16,17,18]. Therefore, it is essential to mine genes conferring blast resistance and heat tolerance (BR-HT).

Previous studies have reported that heat induction could enhance plant resistance to pathogen stresses. On the one hand, HT induction can inhibit the growth of pathogens. For example, cucumber seedlings increased resistance to *Cladosporium cucumerinum* after HT preheating [19]. Susceptible barley varieties enhanced resistance to powdery mildew after preheating [20]. Likewise, melon also strongly resisted *Botrytis cinerea* after HT induction [21]. On the other hand, HT can also induce the expression of resistance genes. The up-regulated expression of yellow rust resistance gene *Yr36* induced by HT enhanced resistance of spring wheat to stripe rust [22]. HT also positively regulated the resistance of *Xa7* [23] and *RP1-D21* [24] in rice and maize, respectively. In addition, the immune crosstalk in plant response to biotic and abiotic stresses deserves further investigation for mining common genes against these stresses. For example, chitinases [25], endogenous hormones [26], reactive oxygen species (ROS) [27], antioxidant enzymes [28], and defense genes [29] play significant roles in plant defense against biotic and abiotic stresses.

With the development of molecular marker technology, quantitative trait loci (QTLs) mapping has become an effective tool to excavate crucial candidate genes to improve rice genetic traits. Both blast resistance and heat tolerance in rice are complicated traits that are controlled by QTLs. Numerous QTLs associated with resistance against blast fungus have been identified from different studies that utilized doubled haploid (DH) [30,31], recombinant inbred lines (RILs) [32,33,34], F2:3 lines [35,36,37], and backcross lines (BILs) [38,39,40] in rice. Because leaf blast is easily evaluated at the seedling stage, which is also crucial for subsequent growth and development, most of these studies chose the seedling stage [41] However, many studies employed the evaluation index of seed set rate or grain quality to identify heat-tolerant QTLs at the reproductive stage (flowering or tassel period), where rice is the most sensitive to HT stress [42,43,44,45,46,47,48]. In addition, many factors in QTL mapping restrict the application of QTLs in practical breeding. These factors include mapping population, molecular marker density, test environment, rice growth stage, etc., which can cause large interval distances, a low accuracy confidence interval (CI), and inconsistent genetic characteristics in QTLs [49,50,51,52].

Goffinet and Gerber [52] developed a computational statistical technique that systematically integrates numerous QTLs from different studies to obtain “consistent” QTLs, namely meta-QTLs, based on the Akaike information criterion (AIC). Meta-QTLs have smaller intervals and higher CI consistency, which is beneficial for mining target genes that can be used for marker-assisted selection (MAS) breeding. Meta-analysis has been applied in multiple species. For instance, Wang et al. [53] collected QTLs for six agriculturally crucial traits (yield, plant height, ear height, leaf angle, stay-green, and maize rough dwarf disease resistance) in maize and then obtained 113 meta-QTLs through meta-analysis. Yin et al. (2017) [54] conducted a meta-analysis for 182 plant-height QTLs in soybean and obtained meta-QTLs with 0.09–5.07 Mb intervals. Islam et al. (2019) [49] implemented a meta-analysis for salt-tolerant QTLs in rice and identified a total of 11 meta-QTLs with higher phenotypic contribution rates, from which candidate salt-tolerant genes were screened out. In addition, RNA-sequencing (RNA-seq) technology is also beneficial for mining vital resistance/tolerance genes [55]. Previous studies have integrated meta-QTLs and RNA-seq to identify key genes for target traits. For example, Kong et al. (2020) [56] screened out significant candidate genes from 418 DEGs located on low-temperature tolerance meta-QTL regions by integration analysis of meta-QTLs and RNA-seq. Likewise, Delfino et al. (2019) [57] screened out 272 important genes, of which 78 were involved in regulating gene expression, signal transduction, growth and development in grape by the same method. However, there are few reports regarding the integration analysis of meta-QTLs and RNA-seq to mine BR-HT genes. In this study, we respectively implemented meta-analyses for published blast-resistant and heat-tolerant QTLs to identify BR-HT meta-QTLs. Combining DEGs from Onaga et al. (2017) [5], we dug out significant BR-HT candidate genes from the above meta-QTL regions.

## 2. Materials and Methods

### 2.1. Bibliographic Collection and Significant Data Summary

We conducted an exhaustive bibliographic collection of papers published from 1994 to 2021 and compiled significant information on QTLs pertaining to blast resistance and HT tolerance in rice. This important information included parent population, mapping-population type, population size, mapping method, QTL number, logarithm of odds (LOD), phenotypic variance (*R*^2^), flanking markers and physical interval of target QTLs. In addition, the QTLs without CIs were calculated according to the published calculation formula [58,59], as follows:

CI = 530/(*N* × *R*^2^) (1)

CI = 163/(*N* × *R*^2^) (2)

where *N* represents the size of mapping population, and *R*^2^ is for the phenotypic variance of target QTLs. Equation (1) is applicable to BILs, DH and F2 populations, and Equation (2) is applicable to RILs.

### 2.2. Meta-QTL Analysis

The physical positions of flanking markers along with target QTLs were determined by Gramene Marker Search (https://archive.gramene.org/db/markers/marker_view, accessed on 9 January 2022). Secondly, we prepared two separate input files (map file and qtl file) in txt format for each study. Following that, a consensus map was constructed, and all the maps with the markers and original QTLs were iteratively projected on a reference map (rice physical map) by Biomercatorv4.2 (https://sourcesup.renater.fr/projects/biomercator/, accessed on 9 January 2022) [60,61]. Meta-QTLs of individual chromosomes were determined based on AIC [52]. Meta-QTLs projected by at least two initial QTLs were selected as results with high reliability, which were left for subsequent analysis [48,50,55]. Then, we utilized Mapchart software (https://www.wur.nl/en/show/Mapchart.htm, accessed on 9 January 2022) [62] to output the vector map of the consensus map. According to the physical positions and sequences of markers flanked at meta-QTL regions, the physical distances of meta-QTL intervals and adjacent genes were determined by NCBI BLAST alignment.

### 2.3. Difference Analysis of RNA-Seq Data

Onaga et al. (2017) [5] provided transcriptome data on CO and LT at seedling stage, which were inoculated with *M. oryzae* isolate, TAN211.16, after preheating 7 days under 35 °C (this treatment was namely 35 °C + *M. oryzae*). The two cultivars both showed stronger resistance to rice blast under the above treatment in marked contrast to 28 °C + *M. oryzae*. There were 6454 DEGs from CO and 5666 DEGs from LT under 35 °C + *M. oryzae* in supplement files.

### 2.4. Integration Analysis of Meta-QTLs and RNA-Seq

A Venn diagram was used to obtain the common genes of meta-QTL interval genes and DEGs from RNA-seq. Via the singular enrichment analysis (SEA) of AgriGo (http://bioinfo.cau.edu.cn/agriGO/analysis.php, accessed on 9 January 2022), Go terms of target genes were obtained, and then, based on FDR (false discovery rate) <0.05, GO enrichment analysis were conducted. The top GO items were visualized by ggplot2 and GOplot R software packages [63].

## 3. Results

### 3.1. Compilation and Characterization of QTL Studies Regarding Blast Resistance in Rice

We updated the collection of blast-resistant QTLs in rice in our previous research (783 blast-resistant QTLs from 43 publications) [64]. A total of 839 blast-resistant QTLs in rice were collected from 51 publications in this study (Table S1). These studies used different parent lines, population size, marker type and mapping method. The overwhelming majority of those studies employed resistant and susceptible cultivars as parent lines. Furthermore, the type of mapping population included RIL, DH, BIL, F2 and F3. The size of the assayed population ranged from 63 to 587. In addition, the mapping methods included interval mapping (IM), composite interval mapping (CIM), single marker analysis (SMA), inclusive composite interval mapping (ICIM), analysis of variance (ANOVA), multiple interval mapping (MIM), simplified composite interval mapping (sCIM), mixed linear model (MLM), and generalized linear mode (GLM). The number of identified QTLs ranged from 1 to 83 in those studies.

### 3.2. Meta-Analysis Results for Blast-Resistant QTLs in Rice

**Figure 1:**
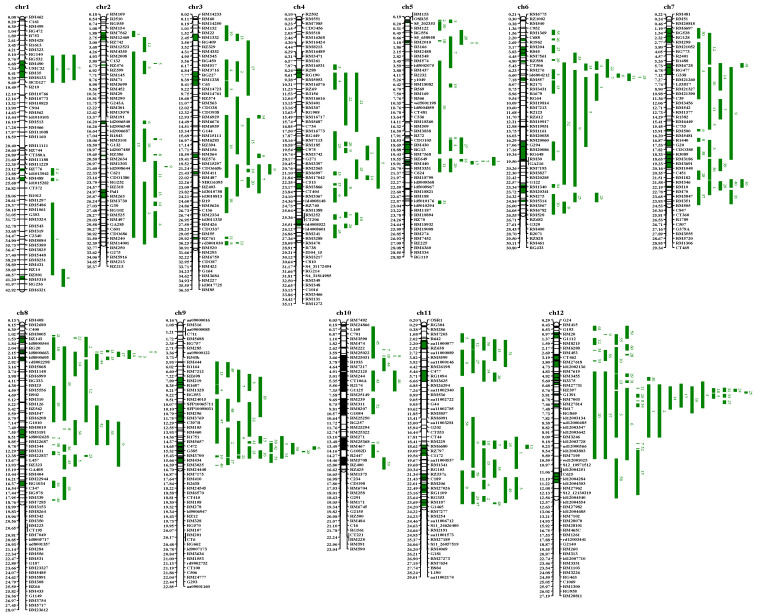
“Consensus” QTL map related to blast resistance in rice. Chr: chromosome; The bars represent the chromosomes; The molecular markers are located on the right of chromosomes, the physical distances of those in mega base (Mb) are located on the left of chromosomes; The original QTLs are positioned on the right of molecular markers in the “consensus” map; Fragments with green color represent confidence intervals of meta-QTLs in the chromosomes.

Through meta-analysis of 839 blast-resistant QTLs, the consensus map contained 1706 markers, with an average distance of 0.22 Mb, and a total of 71 blast-resistant meta-QTLs were identified on 12 chromosomes (Figure 1). Of those, 67 blast-resistant meta-QTLs were projected by at least two original QTLs, which would be subsequently analyzed (Table 1). The maximum number of original QTLs projected to individual meta-QTLs was up to 24. The interval distance of these meta-QTLs ranged from 0.04 to 3.12 Mb, with an average value of 1.00 Mb. Additionally, 42 blast-resistant meta-QTLs had an interval distance of ≤1.0 Mb, and 23 meta-QTLs had an interval distance of ≤0.5 Mb. Furthermore, the number of interval genes in 67 blast-resistant meta-QTLs ranged from 9 to 357. A total of 7054 interval genes were obtained from 67 blast-resistant meta-QTLs (Table S3). In addition, 47 cloned blast-resistant genes were found in 23 meta-QTL regions, among which Metaq11-6 contained 9 blast-resistant genes.

### 3.3. Compilation and Characterization of QTL Studies Regarding Heat Tolerance in Rice

We collected 308 heat-tolerant QTLs in rice from 32 studies published from 2002 to 2021 (Table S2). The parent lines, population size, marker type and mapping method differed in these studies. They employed heat-tolerant and heat-sensitive cultivars as parent population. The size of assayed population ranged from 37 to 1027. Moreover, the mapping methods included IM, CIM, SMA, ICIM, etc. in the previous works. The identified heat-resistant QTLs in those studies ranged from 1–53.

### 3.4. Meta-Analysis Results for Heat-Tolerant QTLs in Rice

**Figure 2:**
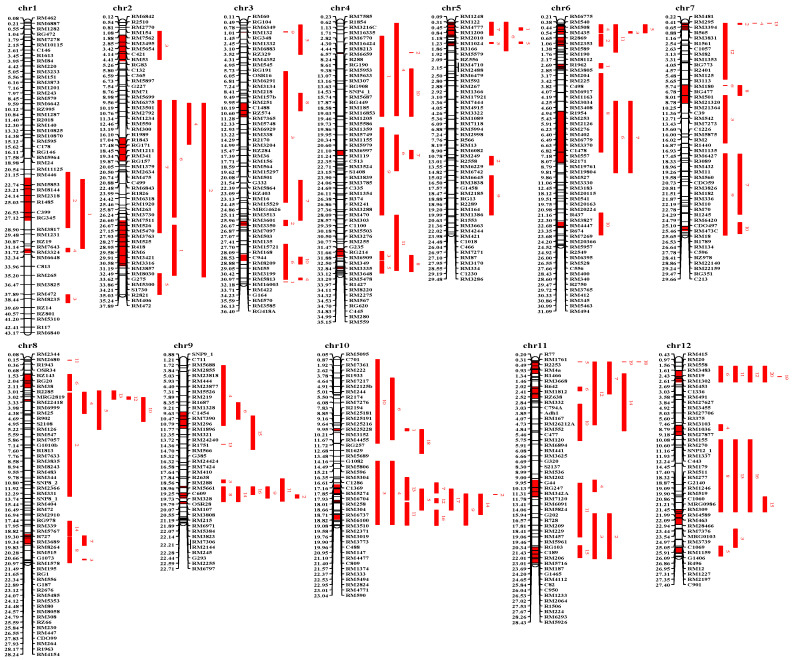
“Consensus” QTL map related to heat tolerance in rice. Chr: chromosome; The bars represent the chromosomes; The molecular markers are located on the right of chromosomes, the physical distances of those in mega base (Mb) are located on the left of chromosomes; The original QTLs are positioned on the right of molecular markers in the “consensus” map; Fragments with red color represent confidence intervals of meta-QTLs in the chromosomes.

With the meta-analysis of 308 heat-tolerant QTLs in rice, 43 heat-tolerant meta-QTLs were detected on 12 chromosomes which contained 1385 markers, with an average distance of 0.27 Mb, (Figure 2). The number of initial QTLs projected to meta-QTLs was 2-7. Among these, 40 heat-tolerant meta-QTLs were derived from at least two initial QTLs (Table 2). The interval distances of these meta-QTLs ranged from 0.03 to 7.17 Mb, with an average value of 1.49 Mb. In addition, ten heat-tolerant meta-QTLs had an interval distance of ≤0.5 Mb, and 20 meta-QTLs had an interval distance of ≤1.0 Mb. Furthermore, the number of interval genes in 40 heat-tolerant meta-QTLs ranged from 6 to 887. A total of 6425 genes were obtained in these meta-QTLs (Table S4). In addition, 21 heat-tolerant genes were found in 10 meta-QTL regions, among which Metah11-1 contained eight heat-tolerant genes.

### 3.5. Integration Analysis Results for Meta-QTLs and RNA-Seq

**Figure 3:**
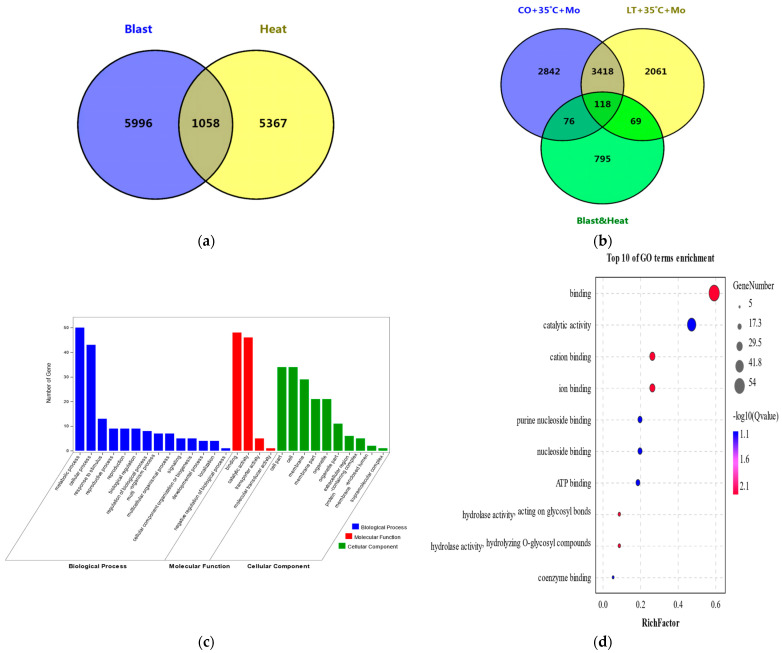
Integration of meta-QTLs and DEGs related to blast resistance and heat tolerance in rice. (**a**) The common genes co-located on meta-QTLs of the two traits; (**b**) the common genes of meta-QTLs and DEGs; (**c**) Go terms of 118 common DEGs co-located on meta-QTLs of the two traits; (**d**) the top 10 GO term enrichment of 118 common DEGs co-located on meta-QTLs of the two traits.

By integrating interval genes from meta-QTLs regarding the two traits, we identified 1058 common genes (Figure 3a and Table S5). There were 6454 DEGs (Table S6) in CO under 35 °C + *M*. *oryzae* and 5666 DEGs (Table S7) in LT under 35 °C + *M*. *oryzae* from Onaga et al. (2017) [5]. By integrating those DEGs, we identified 118 common DEGs (Table S8), which were located on blast-resistant and heat-tolerant meta-QTL regions (Figure 3b). In addition, 118 common DEGs were involved in 14 terms of biological processes (blue column), 4 terms of molecular function (red column), and 10 terms of cellular component (green column) (Figure 3c). Furthermore, assigned to GO enrichment analysis with FDR value < 0.05, the top 5 GO term enrichments were involved in 5 pathways of molecular function, containing 55 genes (Figure 3d and Table S9). These 55 genes were co-located on the common regions of 16 blast-resistant meta-QTLs and 14 heat-tolerant meta-QTLs in 9 chromosomes (Table S10). Among, 34 genes were up-regulated in CO and LT under 35 °C + *M*. *oryzae*, while 21 genes were down-regulated.

## 4. Discussion

In recent years, both HT and rice blast have damaged the growth and development of rice, causing serious losses in rice production. Therefore, breeding new rice varieties with blast resistance and heat tolerance has become an increasingly urgent research task. Over the past few decades, advances in molecular genetics have led to the identification and utilization of QTLs related to yield, abiotic and biotic stress resistance [120,121,122,123,124]. However, the genetic inconsistency of these QTLs hinders their application in MAS breeding [60,124]. Meta-analysis can overcome the limitations of a single study and identify “consistent” QTLs from previous research in different genetic backgrounds and environments. It has been applied in rice [49,60], maize [53] and soybean [51,54]. In addition, compared with genetic maps with lower molecular marker density, physical maps can cover almost all markers from previous genetic maps to the greatest extent. Courtois et al. (2009) [60] used a rice physical map as reference map to identify drought-tolerant meta-QTLs through meta-analysis. Although the genetic map is not consistent with the physical map, the low recombination rate of rice mainly affects the region around the centromere, and the inactive region of rice recombination is limited to a small interval [125]. Therefore, the physical map of rice was used as the reference map in this study. Except for some QTLs with lost data, most QTLs can be anchored to the physical map to minimize the loss of data and increase the number of QTLs available for meta-analysis, which would help improve the accuracy of meta-analysis. In addition, Ballini et al. (2008) [124] conducted a meta-analysis of 347 blast-resistance QTLs of rice in 18 papers, and the results demonstrated that the average interval distance of meta-QTLs was 3.3 Mb, and the average number of original QTLs mapped to individual meta-QTLs was around 1.9. In this study, we conducted a meta-analysis of blast-resistance 839 QTLs from 51 studies in the literature published from 1994 to 2021. The results indicated that the average interval distance of meta-QTLs was 1.00 Mb, and individual meta-QTLs were averagely obtained from 4.8 original QTLs. Likewise, compared with Raza et al. (2020) [126], the average interval distance of heat-tolerance meta-QTLs was smaller in this study. Besides, 47 blast-resistant and 21 heat-tolerant genes published were found in meta-QTL regions (Table 1 and Table 2), such as *Pi27*(t) [65], Pi-Da(t) [66], *Pi14* [67], *Pi16*(t) [68], *Pid1*(t) [68], *OsHsfA7* [104], *OsHsfC1a* [104], *OsWRKY11* [105], *OsGR2* [106], *OsRb1* [107] and *OsHSP58.7* [112]. Therefore, this study not only narrowed the confidence interval, but also improved the credibility of meta-QTLs related to these two traits, which would lay a foundation for further mining of crucial resistant genes.

Previous studies have conducted integrating analysis of RNA-seq and meta-QTLs to screen out key candidate genes for target traits such as cold tolerance in rice [56], seed storage components of soybean [51] and veraison time in grapevine [57]. Although confidence intervals of the above meta-QTLs were further narrowed, the meta-QTLs still contained a large number of genes in this study. Onaga et al. (2017) [5] found that rice varieties CO and LT displayed complete resistance to blast with 35 °C preheating treatment in marked contrast to 28 °C, demonstrating that HT preheating could enhance rice defense against blast fungus. In our study, the RNA-seq data was downloaded from Onaga et al. (2017) and was analyzed with blast-resistant and heat-tolerant meta-QTLs. Totally, we identified 118 common genes, which not only were positioned on meta-QTL regions related to these two traits, but also responded to rice blast and heat stresses. Furthermore, based on GO term enrichment, 55 significant candidate genes were selected (Table S10). Among them, 34 genes were up-regulated, while 21 genes were down-regulated under 35 °C and blast stresses.

To identify blast-resistant and heat-tolerant genes, we preferred to choose genes that were published to be involved in blast resistance or heat tolerance in the previous studies. Consequently, 24 significant BR-HT candidate genes were selected in Table 3. To withstand various stresses, plants have evolved a battery of complicated immune system to protect themselves from various stresses. Especially, PTI (PAMPs triggered immunity) and ETI (effector triggered immunity) play vital roles in defensing against pathogens. Among defense mechanisms, defense response genes (such as chitinases), and resistance (R)-genes are most significant. Five genes, including *C10923*, *OsChib3H-c*, *C10150*, *Os11g0701500*, and *Os11g0702100*, belong to glycosyl hydrolase. Chitinases are just one kind of glycosyl hydrolase. *OsChib3H-c* was co-located on Metab11-6 and Metah11-4 and up-regulated with 3.25–3.36 fold change in CO and LT under 35 °C + *M. oryzae*. *OsChib3H-c* had been identified as a novel chitinase gene, which could enhance resistance to sheath blight pathogen in rice [127]. Interestingly, plant hormones and abiotic stresses also regulated the expression and activity of chitinases [128,129,130,131]. *OsChib3H-c* was also up-regulated to respond to heat [132], drought [133] and jasmonic acid [131]. Thus, we inferred *OsChib3H-c* could confer tolerance to both blast fungus and heat stress. Likewise, it was found that *C10150* [132,134], *C10923* [132,133], *Os11g0702100* [132], and *Os11g0701500* [134,135] were up-regulated to respond to pathogen and abiotic stresses. 

According to the structure characterizes of proteins encoded by R genes [136], nine genes, including *OsJAMyb*, *OsWRKY59*, *OsMYBAS1*, *Pi-k*, *WAK124*, *OsiWAK1*, *WAK3*, *OsRLCK198*, and *SDK6*, belong to resistant gene analogues. *OsJAMyb* is an R2R3 MYB transcription factor, which was located in the same region of Metab11-6 and Metah11-4 and was up-regulated with 5.39–4.98 fold change in CO and LT under 35 °C + *M. oryzae*. It had been reported that *OsJAMyb* overexpressed in Suyu variety to enhance rice defense against blast, suggesting that *OsJAMyb* was involved in resistance to rice blast [98]. *Pi-k* [95], a resistant gene, was also up-regulated with 2.28–2.65 fold change in CO and LT under 35 °C + *M. oryzae*. Besides, WAK kinases (wall associated kinases) are cell wall-associated receptor kinases and had been found to be involved in pathogen resistance and abiotic stress tolerance of rice [99]. *OsWAK1* (*Os11g0690066*) was co-located in Metab11-6 and Metah11-4 regions but not differentially expressed in rice under 35 °C + *M. oryzae*, but *WAK124*, *OsiWAK1*, and *WAK3* were all up-regulated in CO and LT under 35 °C + *M. oryzae.* Previous studies had found that rice resistant genes could be induced to express by preheating [7,137]. For instance, *TaRPM1* [138] under HT stress was up-regulated 6 folds higher than that under normal temperature, which actively regulated the resistance of wheat to wheat stripe rust. *CaWRKY40* had a positive regulatory effect on the single stress of HT and Fusarium wilt [29]. Further studies showed that the interaction between *CaWRKY40* and *CaWRKY6* in pepper regulated the resistance to bacterial blight under HT [139]. Under hormone induction, *TaWRKY70* [137], *TaWRKY49* [140] and *TaWRKY62* [140] participated in the regulation of wheat resistance to the dual stress of HT and stripe rust. Thus, *OsJAMyb, Pi-k, OsWAK124, OsiWAK1*, and *WAK3* could be up-regulated by preheating to enhance CO and LT defense against *M. oryzae.*

On other hand, when plants are suffering from abiotic and biotic stresses, a change in redox state controlled by oxidordeuctases is a common outcome, due to the production and accumulation of reactive oxygen species (ROS) [141,142]. Eight genes were related to oxidordeuctases, including *OsMT2b*, *OsTPS3*, *OsNDB3*, *OsHI-LOX*, *OsLOX8*, *ACLA3*, *OsACLA-2* and *OsGS2*. It depends on the level of ROS to determine whether it will be a defensive or destructive molecule [142,143]. Signal transduction pathways can regulate the level of ROS to protect plants from adverse effects of ROS [142]. In the present study, the metallothionein encoded by *OsMT2b* was co-located on Metab5-1 and Metah5-1 regions and that was down-regulated in CO and LT under 35 °C + *M. oryzae*. Previous study illustrated that *OsMT2b* was down-regulated by the small GTPase OsRac1 in rice to scavenge ROS to increase resistance to bacterial blight and blast fungus [71]. *OsHI-LOX* is a chloroplast-localized type lipoxygenase gene in rice. A previous study had found that *OsHI-LOX* participated in insect-induced JA synthesis and enhanced resistance to BPH (brown planthopper) by scavenging BPH-induced H_2_O_2_ [144]. Likewise, *OsTPS3* (caryophyllene synthase) [145], *OsACLA-2* (ATP-citrate lyases) [146] and *OsGS2* (glutathione synthetase) [147] had been reported to negatively regulate cell death and disease resistance in rice. Thus, *OsMT2b, OsHI-LOX, OsTPS3*, *OsACLA-2* and *OsGS2* could balance the ROS level to defend against blast and HT stresses in CO and LT under 35 °C + *M. oryzae*.

Another one, *OsUBC6,* encoding one of the ubiquitin-conjugating enzymes, E2, was also up-regulated with 2.44–2.62 fold change in CO and LT under 35 °C + *M. oryzae*. Previous research showed that *OsHTAS,* encoding a ubiquitin ligase, interacted with components of the ubiquitin/26S proteasome system to enhance heat tolerance through modulation of hydrogen peroxide-induced stomatal closure [110]. Therefore, *OsUBC6* was inferred to play a role in responding to blast and HT stresses through interaction with components of the ubiquitin/26S proteasome system. 

Overall, integration analysis of meta-QTLs and RNA-seq provided new insight for further screening of candidate genes conferring both blast resistance and heat tolerance in rice. Of course, the genes identified in meta-QTLs should be further investigated.

## 5. Conclusions

In this study, 67 blast-resistant and 40 heat-tolerant meta-QTLs in rice were obtained from 839 and 308 QTLs, respectively. Combined with RNA-seq and GO enrichment analysis, 24 significant genes were mined, which would be the gene resources for functional verification and rice breeding with double tolerance.

## Figures and Tables

**Table 1:** 67 blast-resistant meta-QTLs in rice.

Meta-QTL	Flanking Makers	95% CI (Mb)	Interval Distance (Mb)	Original QTLs	Interval Genes	Published Blast-Resistance Gene
Metab1-1	RM490-RM8133	6.68–9.39	2.71	3	345	*Pi27*(t) [65]
Metab1-2	id1013754-RM2318	23.73–24.14	0.41	2	54	
Metab1-4	RM414-RM14	40.76–41.36	0.61	2	112	
Metab2-1	RG634-RM5654	2.14–3.42	1.28	2	187	*Pi-Da*(t) [66]
Metab2-2	RM492-RM2468	7.29–7.42	0.14	2	11	*Pi14* [67], *Pi16*(t) [68]
Metab2-3	id2006540-RM7426	16.20–16.68	0.48	2	34	
Metab2-4	id2008644-RG25	21.60–21.77	0.17	4	10	*Pid1*(t) [69]
Metab2-5	RM5470-RM6122	27.15–28.44	1.28	4	143	
Metab2-6	GA285-RM6424	29.59–29.63	0.04	4	9	
Metab3-1	RM1332-RG409	2.45–3.50	1.04	2	182	
Metab3-2	C63-RZ574	8.41–10.60	2.19	4	343	
Metab3-3	RM411-RM487	21.43–22.02	0.59	4	52	
Metab3-4	id3010813-R19	24.21–24.60	0.39	7	30	
Metab3-5	RM3684-RM227	34.62–34.93	0.31	3	59	
Metab4-1	RM16531-RM5953	7.98–9.39	1.41	4	62	
Metab4-2	G271-RM2565	20.34–20.93	0.59	5	87	
Metab4-3	id4008148-RM1388	24.36–25.22	0.87	4	138	
Metab4-4	id4008601-RM241	26.12–27.04	0.93	3	138	
Metab4-5	RG214-RM348	31.85–32.84	0.99	5	166	*Pi45*(t) [70]
Metab5-1	RG556-S5_658958	0.45–0.66	0.21	3	40	*OsMT2b* [71]
Metab5-2	id5002075-RM437	3.59–3.88	0.29	4	35	
Metab5-3	RZ649-C624	19.61–21.43	1.83	5	218	*Pi10* [72]
Metab5-4	id5009818-id5010176	22.44–22.87	0.43	4	70	
Metab5-5	id5010294-RM1187	23.04–23.28	0.24	7	33	
Metab6-1	G30-C226A	3.18–3.54	0.36	2	57	
Metab6-2	RM7561-RM2126	4.45–5.91	1.45	2	186	
Metab6-3	RZ144-RZ667	6.72–6.93	0.21	5	32	*Pi8* [73], *Pi13*(t) [67]
Metab6-4	RM19779-RM527	9.32–9.86	0.55	11	31	*Pi40* [74], *Pi22* [75]
Metab6-5	RM541-G122	19.51–22.57	3.06	3	269	
Metab6-6	RG778-G329	26.24–27.61	1.37	3	174	
Metab6-7	R2071-RG653	28.70–29.03	0.33	4	58	*Pitq1* [76]
Metab7-1	RG528-RM21052	1.54–3.77	2.23	2	256	
Metab7-2	RM21260-RM21327	7.27–8.90	1.63	5	121	
Metab7-3	G20-CDO385	17.53–17.82	0.29	5	18	
Metab7-4	RM3691-RM1048	19.23–20.17	0.94	3	104	
Metab7-5	RM346-RM5847	21.05–23.65	2.60	5	357	
Metab7-6	R1789-C507	26.53–26.71	0.18	7	26	
Metab8-1	id8000544-RM6863	1.84–2.01	0.17	3	11	
Metab8-2	id8000695-rd8002298	2.18–2.92	0.73	3	42	*Pi-36* [77]
Metab8-3	RG333-RM5556	4.11–4.59	0.48	9	54	
Metab8-4	RM126-RM6208	5.22–5.79	0.57	10	88	*Pi-42*(t) [78]
Metab8-5	GA408-RM339	16.57–17.95	1.38	8	92	*Pi-11* [79]
Metab8-6	RM342-RM223	19.96–20.65	0.69	4	65	
Metab8-7	id8005717-RM284	20.85–21.15	0.29	5	30	
Metab8-8	RM308-RZ66	24.79–25.59	0.80	3	100	*pi-55*(t) [80]
Metab9-1	R1687-SFP10098031	8.35–10.10	1.75	6	101	*Pi5* [81], *Pi-15* [82], *pi-56* [83]
Metab9-2	RM105-RM434	12.55–15.66	3.12	6	301	
Metab9-3	RM6570-RM108	18.58–19.30	0.73	8	113	
Metab9-4	RZ12-RG570	19.43–19.95	0.52	9	85	
Metab9-5	CT6-RG662	20.17–20.48	0.31	9	54	
Metab9-6	RM1553-C506	21.00–21.86	0.85	11	140	
Metab10-1	RM2125-G1125	4.89–7.34	2.44	6	127	
Metab10-2	RM25149-G1084	7.57–10.64	3.07	2	155	
Metab11-1	RZ638-RM5599	2.52–3.83	1.30	5	147	
Metab11-2	aa11002340-RM536	7.26–8.99	1.74	4	125	*Pi-y*(t) [84], *LHCB5* [85]
Metab11-3	RM6680-RG103	19.08–20.80	1.72	7	181	*Pi-7* [86], *Pi-34* [87], *Pi-38* [88]
Metab11-4	RG1109-RM7277	23.62–24.68	1.06	3	82	*Pi-44*(t) [89]
Metab11-5	RM27154-RM4069	25.23–26.67	1.44	3	123	*Pi54* [90], *Pi-43*(t) [78], *Pi-47*(t) [91]
Metab11-6	RM7654-L190	27.67–28.76	1.09	6	91	*Pik-m* [92], *Pi-46*(t) [93] *Pi-hk1*(t) [94], *Pi-k* [95], *Pi-1* [96], *Pi-18* [97], *Pi-lm2* [76], *OsJAMyb* [98], *OsWAK1* [99]
Metab12-1	G1112-RM6288	1.27–2.20	0.92	4	147	
Metab12-2	RM3455-R3375	4.92–5.61	0.69	5	49	
Metab12-3	G1391-RM7003	5.81–6.78	0.97	2	44	*Pi-6* [100], *Pi-h-1*(t) [101], *Pi-tq6* [76]
Metab12-4	id12003144-id12003547	7.92–8.82	0.90	2	41	*Pi-20* [102], *Pi-21*(t) [97], *Pi-157*(t) [72]
Metab12-5	id12003728-id12003803	9.18–9.54	0.36	2	27	
Metab12-6	C625-id12004303	11.06–11.22	0.16	24	11	
Metab12-7	RM27982-id12004685	12.63–13.06	0.43	5	20	*Pi-ta2* [95], *Pi-19* [103], *Pi-48*(t) [91]
Metab12-8	RM3331-C1069	23.49–25.08	1.59	4	161	

**Table 2:** 40 heat-tolerant meta-QTLs in rice.

Meta-QTL	Flanking Markers	95% CI (Mb)	Map Distance (Mb)	Original QTLs	Interval Gene	Published Heat-Tolerance Gene
Metah1-1	R2159-RM1232	21.70–27.63	5.93	2	671	*OsHsfA7* [104]; *OsHsfC1a* [104]; *OsWRKY11* [105]; *OsGR2* [106]; *OsRb1* [107]; *OsTRBF1* [108]; *OsDfr* [109]; *OsUBC* [110]
Metah1-2	RM6581-RM297	31.50–32.10	0.60	2	101	*Osbht* [17]
Metah2-2	R1989-RM3419	16.10–19.34	3.24	4	253	*OsHsfA5* [104]; *OsClpD1* [111]; *OsHsfA3* [104]
Metah2-3	RM221-RG256	27.61–33.94	6.33	4	887	*OsHSP24.1* [112]; *RCTU1* [113]
Metah3-1	RM3372-RM22	1.46–1.52	0.06	2	13	
Metah3-2	RM7365-RM338	11.28–13.22	1.94	2	237	
Metah3-3	RM15721-RM15759	27.70–28.31	0.61	2	84	
Metah3-4	RM1352-RM143	32.35–33.19	0.84	2	149	*OsHsfA2e* [114]
Metah4-1	RM16424-RM8213	4.30–4.44	0.14	3	15	
Metah4-2	RG449-RM185	17.87–18.58	0.71	4	59	*HTS1* [115]; *eIF3h* [116]
Metah4-3	G235-RM348	31.47–32.65	1.18	2	191	
Metah4-4	RM2799-RM2275	34.14–34.32	0.18	2	31	
Metah5-1	RM153-RZ556	0.19–2.09	1.90	3	264	
Metah5-2	RM1366-R1838	2.92–3.31	0.39	4	48	
Metah6-1	RM4332-RM190	0.72–1.76	1.04	5	155	
Metah6-2	RM8112-RM584	2.17–3.42	1.24	2	207	
Metah6-3	RM2615-RM4128	5.96–6.64	0.69	2	73	
Metah6-4	RM3183-RM20155	12.45–19.61	7.17	3	392	*OsMSRB1.1* [117]
Metah7-1	RM192-RM3831	0.26–1.16	0.91	4	132	
Metah7-2	RM21320-C39	8.78–11.36	2.58	3	131	
Metah7-3	RZ978-RM7601	28.41–29.04	0.63	3	97	
Metah8-1	RM8018-RM6999	2.17–3.98	1.82	5	177	
Metah8-2	RM547-RM6838	5.59–5.85	0.26	7	33	
Metah8-3	RM256-RZ66	24.27–25.67	1.40	3	180	*hsp82A* [118]
Metah9-1	RM5526-RM7364	7.31–9.56	2.25	4	93	*OsHTAS* [119]
Metah9-2	RM410-R2638	17.64–17.84	0.19	2	24	
Metah9-3	RM6570-RM553	18.58–19.32	0.75	4	116	*OsHSP58.7* [112]
Metah9-4	OSR28-RM107	19.79–20.07	0.28	4	43	
Metah10-1	RM1126-RM25228	9.70–9.95	0.25	4	12	
Metah10-2	RM5620-RM5373	17.40–18.73	1.32	3	161	
Metah10-3	RM6132-RM6100	18.79–18.82	0.03	3	6	
Metah10-4	RM2371-C488	19.58–19.96	0.38	6	53	
Metah10-5	RM1374-RM228	21.57–22.24	0.67	6	119	
Metah11-1	R77-R642	0.20–2.02	1.82	3	290	
Metah11-2	C1350-RM5704	3.81–5.48	1.66	5	160	
Metah11-3	RM287-RM5349	16.77–19.18	2.42	2	166	
Metah11-4	RM27234-RM6293	26.10–28.26	2.17	4	237	
Metah12-1	RM3483-RM6296	1.61–3.20	1.59	5	212	
Metah12-2	RM27877-RM270	9.18–10.60	1.42	4	81	
Metah12-4	RM4585-R496	26.13–26.86	0.73	2	72	

**Table 3:** 24 significant candidate genes in the top 5 enrichment GO terms.

RAP-ID	Gene Symbol	Locus Name	Function Annotation	Log2^foldchange^	
				CO	LT
Os11g0700900	*C10923*	LOC_Os11g47500.1	Glycosyl hydrolase, putative, expressed	6.62	6.98
Os08g0508800	*OsHI-LOX*	LOC_Os08g39840.1	Lipoxygenase, chloroplast precursor, putative, expressed	6.08	5.58
Os11g0702100	*chitinases*	LOC_Os11g47600.1	Glycosyl hydrolase, putative, expressed	5.47	6.35
Os11g0701500		LOC_Os11g47560.1	Glycosyl hydrolase, putative, expressed	5.43	3.30
Os11g0684000	*OsJAMyb*	LOC_Os11g45740.1	MYB family transcription factor, putative, expressed	5.39	4.98
Os08g0509100	*OsLOX8*	LOC_Os08g39850.1	Lipoxygenase, chloroplast precursor, putative, expressed	4.21	3.53
Os11g0701400	*C10150*	LOC_Os11g47550.1	Glycosyl hydrolase, putative, expressed	4.17	2.71
Os08g0141400	*OsNDB3*	LOC_Os08g04630.1	External NADH-ubiquinone oxidoreductase 1, mitochondrial precursor, putative, expressed	4.01	4.18
Os11g0660500	*OsTCTP*	LOC_Os11g43900.1	Translationally-controlled tumor protein, putative, expressed	3.80	2.88
Os11g0701000	*OsChib3H-c*	LOC_Os11g47510.1	Glycosyl hydrolase, putative, expressed	3.25	3.36
Os11g0691500	*WAK3*	LOC_Os11g46900.1	Wall-associated receptor kinase 3 precursor, putative, expressed	3.01	3.80
Os09g0321900	*OsUBC6*	LOC_Os09g15320.2	Ubiquitin-conjugating enzyme, putative, expressed	2.44	2.62
Os11g0691100	*OsiWAK1*	LOC_Os11g46860.1	Wall-associated receptor kinase-like 4 precursor, putative, expressed	2.29	3.02
Os11g0689100	*Pi-k*	LOC_Os11g46210.1	NB-ARC domain containing protein, expressed	2.28	2.65
Os12g0266200	*WAK124*	LOC_Os12g16540.1	OsWAK124-OsWAK receptor-like protein OsWAK-RLP, expressed	2.11	4.28
Os11g0700500	*OsMYBAS1*	LOC_Os11g47460.1	MYB family transcription factor, putative, expressed	2.09	2.39
Os04g0631800	*SDK6*	LOC_Os04g53994.1	Kinase, putative, expressed	2.08	3.69
Os11g0684100	*OsWRKY59*	LOC_Os11g45750.2	WRKY protein, expressed	−2.04	−2.21
Os06g0165500	*OsRLCK198*	LOC_Os06g06960.1	S-locus-like receptor protein kinase, putative, expressed	−2.06	−2.41
Os11g0693800	*ACLA3*	LOC_Os11g47120.1	DEFL48-Defensin and Defensin-like DEFL family, expressed	−2.24	−3.01
Os11g0696200	*EDT1; OsACLA-2*	LOC_Os11g47330.1	ATP-grasp domain containing protein, expressed	−2.32	−2.87
Os08g0139700	*OsTPS3*	LOC_Os08g04500.1	Terpene synthase, putative, expressed	−2.51	−5.75
Os05g0111300	*OsMT2b*	LOC_Os05g02070.2	Metallothionein, expressed	−4.32	−2.22
Os12g0263000	*OsGS2*	LOC_Os12g16200.1	Glutathione synthetase, chloroplast precursor, putative, expressed	-4.96	-3.19

## Data Availability

Not applicable.

## Supplementary Materials

The following are available online at https://www.mdpi.com/article/10.3390/genes13020224/s1, Table S1: Information of new publications on blast-resistant QTL mapping in rice, Table S2: Significant information of 32 previous studies related to heat-tolerance QTL mapping, Table S3: Interval genes in 67 blast-resistant meta-QTLs, Table S4. Interval genes in 40 heat-tolerant meta-QTLs, Table S5: Commen genes from blast-resistant meta-QTLs and heat-tolerant meta-QTLs, Table S6: Differently expressed genes (Gene ID converted from MSU to RAP type) in CO under 35 °C and blast stresses, Table S7: Differently expressed genes (Gene ID converted from MSU to RAP type) from LT under 35 °C and blast stresses, Table S8: Commen genes from meta-QTLs and DEGs in rice, Table S9: Genes in the top 5 enrichenment GO terms, Table S10: Significant information of 55 genes in the top 5 enrichment GO terms.
