# Supplementary material for: Selection of Candidate Genes Conferring Blast Resistance and Heat Tolerance in Rice through Integration of Meta-QTLs and RNA-Seq
Source: Genes (Basel). 2022 Jan 25;13(2):224. doi: 10.3390/genes13020224 (PMC8871662; doi:10.3390/genes13020224)
Supplement: Supplementary file 1 [file genes-13-00224-s001.zip › Supplementary Table S1-S2 - final.pdf]

Supplementary Material

Table S1. Information of new publications on blast-resistant QTL mapping in rice*.

Mapping Population	Population Type	Population Size	Detection QTL Method	Number of QTL	References
Hokkai188×Danghang-Shali	F2	190	IM	2	Nguyen et al., 2006 [1]
SHZ-2×TXZ-13	BILs	244	SMA	3	Liu et al., 2011 [2]
Nipponbare×93-11	RILs	259	CIM	4	Yang et al., 2013 [3]
Miyazakimochi×Bikei 22	RILs	158	CIM	2	Ishihara et al., 2014 [4]
DGWG×RR9	RILs	175	SMA, CIM	6	Liu et al., 2015 [5]
DGWG×RR20	RILs	224	SMA, CIM	22	
Akhanaphou×Leimaphou	RILs	103	ICIM	2	Aglawe et al., 2017 [6]
Landraces	Landraces	314	GLM, MLM	32	Lin et al., 2018 [7]
INGR15002×BPT 5204	F2/F3	188	ICIM	4	Devi et al., 2020 [8]
INGR15002×CO-39	F2/F3	188			
INGR15002×BPT5204	BILs	188			

* Update from our previous research publication. RILs: recombinant inbred lines; DH: doubled haploid; BILs: backcross lines; IM: interval mapping; CIM: composite interval mapping; SMA : single marker analysis; MLM: Mixed linear model; GLM: generalized linear model; ICIM : inclusive composite interval mapping.

Table S2. Information of 32 publications on heat-tolerant QTL mapping in rice.

Mapping Population	Populati on Types	Population Size	Detection QTL Method	Number of QTL	Reference
IR64×Azucena	DH	109	Likelihood ration Chi square test	6	Cao et al. 2002 [9]
Nipponbare×Kasalath	BILs	98	MLM	3	Zhu et al. 2005 [10]
Nipponbare×Kasalath	BILs	98	MLM	9	Zhu et al. 2006 [11]
USSR5×Guangjie 9	BILs	123	MLM	7	Zhao et al. 2006 [12]
Chiyonishiki×Koshijiwase	RILs	107	CIM	4	Tabata et al. 2007 [13]
T219×T226	RILs	202	MIM, Likelihood ration Chi square test	6	Chen et al. 2008 [14]
Zhongyouzao8×Fengjin	RILs	168	MIM	3	Zhang et al. 2008 [15]
Teqing×Yuanjiangpuye	BILs	77	SMA	4	Kui et al. 2008 [16]
Bala×Azucena	RILs	181	MIM	16	Jagadish et al. 2010 [17]
996×4628	RILs	286	CIM	2	Xiao et al. 2011a [18]
996×4628	RILs	286	CIM	2	Xiao et al. 2011b [19]
Xiushui09×IR2061	BILs	240	MLM	4	Cheng et al. 2012 [20]
IR64×N22	BILs	158	CIM	16	Ye et al. 2012 [21]
HE×NW	RILs	178	CIM	12	kobayashi et al. 2013 [22]
OM5930×N22	BILs	310	SMA, IM, CIM	11	Buu et al. 2014 [23]
YJ10-03-01×Shuhui527	F2	1027	MIM	1	Cao et al. 2015 [24]
Shuhui527×6 different indica rice	BILs	131	ANOVA	43	Liu et al.2015 [25]
Nipponbare×Kasalath	BILs	95	CIM	16	Tazib et al.2015 [26]
Tsukushiroman×Chikushi52	RILs	88	CIM	10	Wada et al. 2015 [27]
Sasanishiki×Habataki	CSSLs	37	ANOVA	11	Zhao et al. 2016 [28]
Milyang23×Giza178	F2	96	CIM	2	Ye et al. 2015 [29]

IR64×Giza178	F2	86		4	
IR64×Milyang23×Giza178	BILs	100		5	
N22×IR64	RILs	272	ICIM	5	Ps et al. 2017 [30]
Rejing35×XieB	F2	400	ICIM	3	Zheng et al. 2017 [31]
996×4628	RILs	124	CIM	24	Zhang et al. 2017 [32]
Sasanishiki×Habataki	CSSLs	39	Dunnett's multiple comparison test	12	Zhu et al. 2017 [33]
Tsukushiroman×Chikushi52	RILs	88	Dunnett's multiple comparison test	4	Miyahara et al. 2017 [34]
IAPAR-9×Liaoyan241	RILs	200	ICIM	7	Li et.al. 2018 [35]
Bg90–2×Shuhui527	BILs	400	SMA, IM	53	Udawela et al. 2018 [36]
Mengguandamagu×Shuhui527	BILs	400			
YJ01-201×TianfengB	BILs	586	Substitution mapping	1	Cao et al. 2019 [37]
IRGC102309×9311	BILs	420	CIM	1	Cao et al. 2020 [38]
Cheongcheong×Nagdong	DH	120	CIM	19	Park et al. 2020 [39]
Cheongcheong×Nagdong	DH	120	CIM	2	Park et al. 2021 [40]

CSSLs: chromosome segment substitution lines; ANOVA: analysis of variance; MIM: multiple Interval mapping; sCIM: Simplified composite interval mapping.

Reference

1. Nguyen, T.T.; Koizumi, S.; La, T.; Zenbayashi, K.S.; Ashizawa, T.; Yasuda, N.; Imazaki, I.; Miyasaka, A. *Pi35*(t), a new gene conferring partial resistance to leaf blast in the rice cultivar Hokkai 188. *Theor. Appl. Genet.* **2006**, *113*, 697-704.
2. Liu, Y.; Zhu, X.Y.; Zhang, S.; Bernardo, M.; Edwards, J.; Galbraith, D.W.; Leach, J.; Zhang, G.; Liu, B.; Leung, H. Dissecting quantitative resistance against blast disease using heterogeneous inbred family lines in rice. *Theor. Appl. Genet.* **2011**, *122*, 341-353.
3. Yang, H.; Jia, M.H.; Jia, Y.; Xing, J.; Venu, R.C.; Bellizzi, M.; Yuan, L.; Wang, Z.; Sun, C.; Wang, G.L. Molecular mapping of four blast resistance genes using recombinant inbred lines of 93-11 and nipponbare. *J. Plant Biol.* **2013**, *56*, 91-97.
4. Ishihara, T.; Hayano-Saito, Y.; Oide, S.; Ebana, K.; La, N.T.; Hayashi, K.; Ashizawa, T.; Suzuki, F.; Koizumi, S. Quantitative trait locus analysis of resistance to panicle blast in the rice cultivar Miyazakimochi. *Rice (N Y)* **2014**, *7*, 2.
5. Liu, Y.; Qi, X.; Gealy, D.R.; Olsen, K.M.; Caicedo, A.L.; Jia, Y. QTL analysis for resistance to blast disease in U.S. Weedy Rice. *Mol. Plant Microbe. Interact.* **2015**, *28*, 834-844.
6. Aglawe, S.B.; Bangale, U.; Ramadevi, S.J.; Balija, V.; Pal, B.V.; Kumar, S.S.; Kumar, S.P.; Kumar, S.; Maddamshetty, S.P.; Maganti, S.M. Identification of novel QTLs conferring field resistance for rice leaf and neck blast from an unique landrace of India. *Gene Rep.* **2017**, *7*, 35-42.
7. Lin, H.A.; Chen, S.Y.; Chang, F.Y.; Tung, C.W.; Chen, Y.C.; Shen, W.C.; Chen, R.S.; Wu, C.W.; Chung, C.L. Genome-wide association study of rice genes and loci conferring resistance to *Magnaporthe oryzae* isolates from Taiwan. *Bot. Stud.* **2018**, *59*, 32.
8. Devi, S.J.S.R.; Singh, K.; Umakanth, B.; Vishalakshi, B.; Rao, K.V.S.; Suneel, B.; Sharma, S.K.; Kadambari, G.K.M.; Prasad, M.S.; Senguttvel, P.; Syamaladevi, D.P.; Madhav, M.S. Identification and characterization of a large effect QTL from Oryza glumaepatula revealed *Pi68*(t) as putative candidate gene for rice blast resistance. *Rice (N Y)* **2020**, *13*, 17.
9. Cao, L.; Zhu, J.; Zhao, S.; He, L.; Yan, Q. Mapping QTLs for heat tolerance in a DH population from *indica-japonica* cross of rice (*Oryza sative*). *J. Agr. Biotechnol.* **2002**, *10*, 210-214.
10. Zhu, C.L.; Xiao, Y.H.; Wang, C.M.; Jiang, L.; Zhai, H.Q.; Wan, J.M. Mapping QTL for heat-tolerance at

grain filling stage in rice. *Rice Sci.* **2005**, *12*, 33-38.

11. Zhu, C.L.; Jiang, L.; Zhang, W.W.; Wang, C.M.; Zhai, H.Q.; Wan, J.M. Identifying QTLs for thermo-tolerance of amylose content and gel consistency in rice. *Rice Sci.* **2006**, *20*, 248-252.
12. Zhao, Z.G.; Jiang, L.; Xiao, Y.H.; Zhang, W.W.; Zhai, H.Q.; Wan, J.M. Identification of QTLs for heat tolerance at the booting stage in rice (*Oryza sativa* L.). *Acta Agron. Sin.* **2006**, *32*, 640-644.
13. Tabata, M.; Hirabayashi, H.; Takeuchi, Y.; Ando, I.; Iida, Y.; Ohsawa, R. Mapping of quantitative trait loci for the occurrence of white-back kernels associated with high temperatures during the ripening period of rice (*Oyza sativa* L.). *Breed. Sci.* **2007**, *57*, 47-52.
14. Chen, Q.Q.; Yu, S.B.; Li, C.M.; Mou, T.M. Identification of QTLs for heat tolerance at flowering stage in rice. *Sci. Agr. Sin.* **2008**, *41*, 315-321.
15. Zhang, T.; Yang, L.; Jang, K.F.; Huang, M.; Huang, M.; Chen, W.F.; Zheng, J.K. QTL mapping for heat tolerance of the tassel period of rice. *Mol. Plant Breed.* **2008**, *6*, 867-873.
16. Kui, M.L.; Tan, L.B.; Tu, J.; Lu, Y.X.; Sun, C.Q. Identification of QTLs associated with heat tolerance of Yuanjiang common wild rice (Oryza rufipogon Griff.) at flowering stage. *J. Agr. Biotechnol.* **2008**, *16*, 461-464.
17. Jagadish, K.; Cairns, J.; Lafitte, R.; Wheeler, T.R.; Price, A.H.; Craufurd, P.Q. Genetic analysis of heat tolerance at anthesis in rice. *Crop Sci.* **2010**, *50*, 1633-1641.
18. Xiao, Y.H.; Pan, Y.; Luo, L.H.; Zhang, G.L.; Deng, H.B.; Dai, L.Y.; Liu, X.L.; Tang, W.B.; Chen, L.Y.; Wang, G.L. Quantitative trait loci associated with seed set under high temperature stress at the flowering stage in rice (*Oryza sativa* L.). *Euphytica* **2011**, *178*, 331-338.
19. Xiao, Y.H.; Pan, Y.; Luo, L.H.; Deng, H.B.; Zhang, G.L.; Tang, W.B.; Chen, L.Y. Quantitative trait loci associated with pollen fertility under high temperature stress at flowering stage in rice (*Oryza sativa*). *Rice Sci.* **2011**, *18*, 204-209.
20. Cheng, L.R.; Wang, J.M.; Uzokwe, V.; Meng, L.J.; Wang, Y.; Sun, Y.; Zhu, L.H.; Xu, J.L.; Li, Z.K. Genetic analysis of cold tolerance at seedling stage and heat tolerance at anthesis in rice (*Oryza sativa* L.). *J. Integr. Agr.* **2012**, *11*, 359-367.
21. Ye, C.; Argayoso, M.A.; Redoña, E.D.; Sierra, S.N.; Laza, M.A.; Dilla, C.J.; Mo, Y.; Thomson, M.J.; Chin, J.; Delaviña, C.B.; Diaz, G.Q.; Hernandez, J.E. Mapping QTL for heat tolerance at flowering stage in rice using SNP markers. *Plant Breeding* **2012**, *131*, 33-41.
22. Kobayashi, A.; Sonoda, J.; Sugimoto, K.; Kondo, M.; Iwasawa, N.; Hayashi, T.; Tomita, K.; Yano, M.; Shimizu, T. Detection and verification of QTLs associated with heat-induced quality decline of rice (*Oryza sativa* L.) using recombinant inbred lines and near-isogenic lines. *Breed. Sci.* **2013**, *63*, 339-346.
23. Buu, B.C.; Ha, P.; Tam, B.P.; Nhien, T.T.; Lang, N.T. Quantitative trait loci associated with heat tolerance in rice (*Oryza sativa* L.). *Plant Breed. Biotech.* **2014**, *2*, 14-24.
24. Cao, Z.B.; Xie, H.W.; Nie, Y.Y.; Mao, L.H.; Li, Y.H.; Cai, Y.H. Mappinga QTL (*qHTH5*) for heat tolerance at the heading stage on rice chromosome5 and its genetic effect analysis. *Chin. J. Rice Sci.* **2015**, *29*, 119-125.
25. Liu, Z.; Tang, Q.; Li, F.; Kapila Siri Udawela, U.A.; Xiang, C.; Gao, Y.; Shi, Y. Heat tolerant evaluation and QTL mapping under flowering stage in indica rice. *Mol. Plant Breed.* **2015**, *13*, 16-31.
26. Tazib, T.; Kobayashi, Y.; Koyama, H.; Matsui, T. QTL analyses for anther length and dehiscence at flowering as traits for the tolerance of extreme temperatures in rice (*Oryza sativa* L.). *Euphytica* **2015**, *203*, 629-642.
27. Wada, T.; Miyahara, K.; Sonoda, J.Y.; Tsukaguchi, T.; Miyazaki, M.; Tsubone, M.; Ando, T.; Ebana, K.; Yamamoto, T.; Iwasawa, N.; Umemoto, T.; Kondo, M.; Yano, M. Detection of QTLs for white-back and

basal-white grains caused by high temperature during ripening period in *japonica rice*. *Breed. Sci.* **2015**, *65*, 216-225.

28. Zhao, L.; Lei, J.; Huang, Y.; Zhu, S.; Chen, H.; Huang, R.; Peng, Z.; Tu, Q.; Shen, X.; Yan, S. Mapping quantitative trait loci for heat tolerance at anthesis in rice using chromosomal segment substitution lines. *Breed. Sci.* **2016**, *66*, 358-366.
29. Ye, C.; Tenorio, F.A.; Argayoso, M.A.; Laza, M.A.; Koh, H.J.; Redoña, E.D.; Jagadish, K.S.; Gregorio, G.B. Identifying and confirming quantitative trait loci associated with heat tolerance at flowering stage in different rice populations. *BMC Genet.* **2015**, *16*, 41.
30. Ps, S.; Sv, A.M.; Prakash, C.; Mk, R.; Tiwari, R.; Mohapatra, T.; Singh, N.K. High resolution mapping of QTLs for heat tolerance in rice using a 5K SNP array. *Rice (N Y)* **2017**, *10*, 28.
31. Zheng, J.; Li, X.X.; Su, H.Y.; Zhou, J.J.; Qin, X.J.; Nan, W.B.; Yang, Y.Q.; Zhang, H.M.; Li, X.Y.; Liang, Y.S. Construction genetic map of japonica rice resource Rejing35 and heat-resistance QTL analysis. *J. Nucl. Agr.Sci.* **2017**, *31*, 844-851.
32. Zhang, G.L.; Liao, B.; Tang, W.B.; Chen, L.Y.; Xiao, Y.H. Identifying QTLs for thermo-tolerance of grain chalkiness trait in rice. *Chin. J. Rice Sci.* **2017**, *31*, 257-264.
33. Zhu, S.; Huang, R.; Wai, H.P.; Xiong, H.L.; Shen, X.H.; He, H.H.; Yan, S. Mapping quantitative trait loci for heat tolerance at the booting stage using chromosomal segment substitution lines in rice. *Physiol. Mol. Biol. Plants* **2017**, *23*, 817-825.
34. Miyahara, K.; Wada, T.; Sonoda, J.Y.; Tsukaguchi, T.; Miyazaki, M.; Tsubone, M.; Yamaguchi, O.; Ishibashi, M.; Iwasawa, N.; Umemoto, T.; Kondo, M. Detection and validation of QTLs for milky-white grains caused by high temperature during the ripening period in *Japonica rice*. *Breed. Sci.* **2017**, *67*, 333-339.
35. Li, M.M.; Li, X.; Yu, L.Q.; Wu, J.; Li, H.; Liu, J.; Ma, X.; Jo, S.; Park, D.S.; Song, Y.; Shin, D.; Han, L. Identification of QTLs associated with heat tolerance at the heading and flowering stage in rice (*Oryza sativa* L.). *Euphytica* **2018**, *214*, 70-81.
36. Udawela, U.; Ananda, G.; Karunarathne, S.I.; Sooriyapathirana, S.; Li, F. Development of heat tolerant introgression lines and preliminary quantitative trait loci (QTL) analysis at flowering stage in *Oryza sativa* L. *J. Nat. Sci. Foundation Sri. Lanka* **2018**, *46*, 253-270.
37. Cao, Z.; Tang, X.; Xiao, W.; Mao, L.; Nie, Y.; Li, Y.; Xie, H.; Cai, Y.; Yuan, L. Identification and genetic effect analysis of QTL (*qHTH10*) for heat tolerance at heading and flowering stage of rice. *Mol. Plant Breed.* **2019**, *17*, 2223-2230.
38. Cao, Z.; Li, Y.; Zeng, B.; Mao, L.; Cai, Y.; Wu, X.; Yuan, L. QTL mapping for heat tolerance of chalky grain rate of *Oryza glaberrima* steud. *Chin. J. Rice Sci.* **2020**, *34*, 135-142.
39. Park, J.R.; Yang, W.T.; Kim, D.H.; Kim, K.M. Identification of a novel Gene, *Osbht*, in response to high temperature tolerance at booting stage in rice. *Int. J. Mol. Sci.* **2020**, *21*, 5862.
40. Park, J.R.; Kim, E.G.; Jang, Y.H.; Kim, K.M. Screening and identification of genes affecting grain quality and spikelet fertility during high-temperature treatment in grain filling stage of rice. *BMC Plant Biol.* **2021**, *21*, 263.
